# A Centrifugal Microfluidic Platform Integrating Immunomagnetic Separation and Isothermal Amplification for Rapid and High-Sensitivity Detection of Foodborne Pathogens

**DOI:** 10.3390/bios16060321

**Published:** 2026-06-02

**Authors:** Qingfeng Zheng, Zhun Zhuang, Hua Lei, Jianhan Lin, Hua Yang, Ruibin Hu

**Affiliations:** 1 Institute of Biotechnology, Xianghu Laboratory, Hangzhou 311231, China; zhengqingfeng@xhlab.ac.cn (Q.Z.); sy20243082075@cau.edu.cn (Z.Z.); leihua@xhlab.ac.cn (H.L.); 2 College of Information and Electrical Engineering, China Agricultural University, Beijing 100083, China; 3 Zhejiang Academy of Agricultural Sciences, Hangzhou 310021, China

**Keywords:** foodborne pathogens, immunomagnetic separation, multienzyme isothermal rapid amplification, centrifugal microfluidic chip, point-of-care testing

## Abstract

The contamination by foodborne pathogens posed a significant health threat and huge economic burden. Traditional detection methods were limited by cumbersome and time-consuming procedures, low automation, and reliance on expensive instrumentation, making them inadequate for on-site detection. This paper presented a centrifugal microfluidic chip that integrated sample pretreatment, nucleic acid extraction, amplification reaction, and signal detection. The chip featured an innovative design that combined a bursting valve with the siphon channel and employed a dual-channel configuration for splitting and directing the flow of different reagents, thereby overcoming the instability issue of unintended pre-activation or interruption that often occurred in the cascade design of multilevel siphon channels. Moreover, by synergistically combining with immunomagnetic separation as well as multi-enzyme isothermal rapid amplification, a portable, easy, rapid, high-sensitivity, and low-cost point-of-care testing (POCT) system for foodborne pathogens was developed. Under optimized conditions, the system enabled detection of *Salmonella* in spiked milk samples at 10 CFU/mL in 1 h. The recoveries ranged from 83.22% to 127.60%, with relative standard deviations of ≤13.7%, indicating that this system had great potential for rapid and high-sensitivity detection of foodborne pathogens in resource-limited settings.

## 1. Introduction

Contamination by foodborne pathogens is one of the most severe public health challenges globally. The sudden outbreaks not only severely threatened public health but also resulted in a heavy burden on socioeconomic stability. According to the World Health Organization (WHO), foodborne pathogenic infections resulted in 420,000 deaths worldwide each year. Each year, 110 billion US dollars were lost in productivity and medical expenses resulting from unsafe foods in low-income countries [1]. As the food supply chains became increasingly diverse and complex, contamination by foodborne pathogens could occur at every stage, from production, processing, transportation, to sales [2]. Currently, traditional methods for detecting foodborne pathogens primarily rely on microbial culture, immunological assays, and molecular biological assays represented by polymerase chain reaction (PCR) [3]. However, these methods exhibited non-negligible limitations when dealing with sudden epidemic outbreaks. Microbial culture achieved high accuracy but involved complicated procedures and extremely long consumption times. It generally took 24 to 72 h or even longer to obtain preliminary results, which could hardly meet the needs for rapid traceability and decision-making during outbreaks [4]. Immunological assays [5,6], such as enzyme-linked immunosorbent assay (ELISA) and lateral flow immunoassay (LFIA), were characterized by easy operation, simple equipment, and low cost, making them suitable for on-site rapid screening. Nevertheless, they were vulnerable to non-specific interference from complex food matrices, leading to low sensitivity and poor accuracy. PCR-based molecular biological methods achieved precise identification of pathogens by specifically amplifying target nucleic acid sequences [7,8]. They exhibited high sensitivity and could shorten detection time to a couple of hours. However, they heavily depended on specialized laboratories and large, expensive instruments. In addition, sample pretreatment procedures (such as enrichment, nucleic acid extraction, and purification) were cumbersome and required high professional skills, which restricted their popularization in resource-limited scenarios [9,10]. Thus, traditional detection methods had inherent deficiencies in speed, sensitivity, specificity, portability, and cost, making them inadequate for the current demands of diversified food supply chains, complex contamination scenarios, and instantaneous epidemic prevention and control [11,12].

Among numerous emerging detection technologies, microfluidic chips [13,14,15] have emerged as a critical pathway to achieve point-of-care testing (POCT) of foodborne pathogens [16,17,18,19], capitalizing on their core advantages of miniaturization, integration, and automation. By constructing functional units such as microchannels, microreaction chambers, microvalves, and micropumps [20,21], microfluidic chips could integrate multiple detection procedures onto a single chip, including sample pretreatment, nucleic acid extraction, amplification reaction, and signal detection [22,23]. This integration obviated the need for cumbersome manual operations and facilitated automated detection from sample in to result out, thereby effectively minimizing human error. Moreover, the detection time could be shortened to 1 h or shorter, significantly enhancing detection efficiency. Additionally, microfluidic chips supported parallel detection of multiple samples or simultaneous detection of multiple pathogens, which greatly enhanced detection throughput and met the demands for batch food detection and multi-pathogen screening [24,25]. Existing studies have verified the practicability of microfluidic detection systems. For instance, Xiao et al. established an integrated chip to achieve rapid, sensitive, and automatic detection of *Escherichia coli* and *Salmonella* in only 40 min [26]. However, several technical bottlenecks and practical challenges still existed in the practical popularization and application of microfluidic chips, including the complex structural design and high manufacturing cost of highly integrated chips, the limited pretreatment capacity for complex samples, and the insufficient stability of detection signals for low-concentration samples caused by the restricted signal amplification capability of miniaturized devices. The combination of nucleic acid amplification techniques and microfluidic chips might improve the sensitivity, specificity, and practicality of detection systems, and has become a research hotspot. Unlike traditional PCR, which requires repeated temperature cycling, isothermal nucleic acid amplification techniques enable rapid and efficient amplification of target nucleic acid sequences under a constant temperature, without relying on sophisticated thermal cyclers. With simple equipment requirements, they were more easily integrated into portable detection devices [27,28,29]. Representative examples included loop-mediated isothermal amplification (LAMP) [30,31], recombinase-aided amplification (RAA) [32,33], recombinase polymerase amplification (RPA) [34,35,36], and multienzyme isothermal rapid amplification (MIRA). A further comparison with standard PCR workflows and representative isothermal amplification assays is summarized in Table S1. Among them, MIRA attracted increasing attention in recent years. By simulating biological DNA recombination and repair processes and leveraging the synergistic action of multiple enzymes, MIRA enabled rapid exponential amplification of nucleic acids at a constant temperature of 37–42 °C. Furthermore, the flexible design of specific primers and fluorescent probes ensured accurate target gene recognition with high detection specificity [37,38]. MIRA, combined with lateral flow dipstick (LFD) technology, was reported to detect *Bacillus cereus* in rice samples down to 115 CFU/mL [39]. However, isothermal amplification techniques still present certain limitations in real sample detection. On one hand, the concentration of foodborne pathogens in actual food samples was often low, even below the minimum detection limit of these techniques. On the other hand, impurities in the food matrix (such as fats, proteins, polysaccharides, etc.) generally had an impact on nucleic acid amplification efficiency and caused false positive or negative signals [40]. Immunomagnetic separation technology was featured with high specificity, high enrichment efficiency, simple operation, and short time consumption (usually 20–30 min), which could significantly improve the detection sensitivity of nucleic acid amplification [41,42]. Meanwhile, specific capture could effectively remove interfering substances from the sample matrix, reduce their impact on the amplification reaction, and improve the detection specificity and accuracy. Jin used magnetic separation technology to achieve bacterial enrichment on a finger-actuated microfluidic chip, which could complete multiplex *Salmonella* detection as low as 9 CFU per sample in 45 min [43].

In this study, immunomagnetic separation and enrichment, isothermal nucleic acid amplification, and microfluidic chip technologies were rationally combined to complement their respective advantages, thereby overcoming numerous limitations of conventional detection methods. Specifically, immunomagnetic separation and enrichment were used to achieve the specific capture, isolation, and enrichment of target pathogenic bacteria in samples, so as to address the challenges of undetectable low-concentration contamination and matrix interference. Isothermal nucleic acid amplification enabled efficient amplification of target nucleic acids under constant and rapid reaction conditions, ensuring high sensitivity and specificity of detection; meanwhile, it required no sophisticated equipment and was thus convenient for miniaturized integration. The whole detection procedures, including sample pretreatment, nucleic acid amplification, and signal detection, were integrated onto a single microfluidic chip, realizing the automation, miniaturization, and high-throughput capacity of the detection workflow, which greatly simplified the operation process, reduced the detection cost, and improved the detection efficiency. To achieve stable operation of the multi-step integrated microfluidic chip, we innovatively solved the problem of unintended pre-activation in the cascade design of multilevel siphon channels by combining a bursting valve on the siphon channel. The problem of a single siphon channel being difficult to stably reactivate when transferring different reagents was solved by using a dual-channel diversion method [44,45,46]. In addition, we further developed a portable POCT system for foodborne pathogens with simple operation, rapid detection, high sensitivity, and low cost.

## 2. Materials and Methods

### 2.1. Reagents and Materials

*Salmonella typhimurium* (ATCC 14028) was selected as the target bacterium, while *E. coli* O157:H7 (ATCC 43888), *Staphylococcus aureus* (CICC 10001), *Vibrio parahaemolyticus* (ATCC 17802) and *Listeria monocytogenes* (ATCC 13932) were used as non-target bacterial strains. Polyclonal antibodies against *Salmonella* (20C-CR7100RP, 2.5 mg/mL) were obtained from Biosynth (Staad, Switzerland). Carboxylated magnetic nanoparticles (10 mg/mL, 180 nm, CV ≤ 10%) were purchased from So-Fe Biomedicine (Shanghai, China). 1-Ethyl-(3-dimethylaminopropyl) carbodiimide hydrochloride (EDC·HCl), N-hydroxysulfosuccinimide sodium salt (NHSS), and 2-morpholinoethanesulfonic acid (MES) were purchased from Aladdin (Shanghai, China). Phosphate buffered saline (PBS, 10 mmol/L, pH 7.4) was acquired from Sangon Biotech (Shanghai, China). Skim milk powder was obtained from neoFroxx GmbH (Einhausen, Germany). Sucrose and bovine serum albumin (BSA) were purchased from Sigma-Aldrich (Taufkirchen, Germany). LB nutrient agar was supplied by Aoboxing (Beijing, China). Isopropanol and dimethyl silicone oil were obtained from Macklin (Shanghai, China). Skimmed milk was purchased from the supermarket. Hydrophilic and hydrophobic modification reagents were purchased from Mesobiosystem (Wuhan, China). Nucleic acid release buffer and lyophilized microspheres of MIRA (containing primers and fluorescent probes) were custom synthesized by Ampu Biotech (Weifang, China). Deionized water used for solution preparation was produced by Millipore Advantage A10 system (Millipore Corporation, Burlington, MA, USA; 18.2 MΩ·cm).

Most electronic hardware used to construct the POCT system was commercially available and could be purchased online, except for custom optical components obtained from Coraytech (Qingdao, China), including twelve condenser lenses (3 mm), four excitation filters (488 nm), and one fluorescence detection filter (520 nm). Supporting components such as the centrifugal tray module and instrument housing were fabricated by 3D printing. 

The PMMA-based microfluidic chip was designed using AutoCAD 2016 software (version R20.1, Autodesk, San Francisco, CA, USA) and manufactured via computer numerical control (CNC) machining by a professional processing factory. Different from multi-layer stacked microfluidic chips, the proposed chip adopts a single-layer structural design, which eliminates the complex multi-layer alignment operation and avoids structural errors caused by layer misalignment. The chip was tightly packaged with transparent pressure-sensitive adhesive (PSA) film. During the bonding process, the adhesive film was fully flattened and laminated slowly to eliminate internal bubbles and prevent microchannel blockage. Residual edge materials were trimmed neatly to ensure the overall structural integrity and flatness of the chip. The microfluidic chip was strictly intended for single-use to eliminate the risk of nucleic acid cross-contamination between different samples.

### 2.2. Preparation of the Immunomagnetic Nanoparticles

100 μL of carboxylated magnetic nanoparticles (10 mg/mL) was washed twice with 1 mL of MES buffer (20 mmol/L, pH 5.5) and then resuspended in 1 mL of freshly prepared MES buffer containing 10 mmol/L EDC·HCl and 5 mmol/L NHSS. Activation reaction of carboxyl groups on the magnetic nanoparticles was performed at 37 °C for 1 h in a thermostatic shaker at 200 rpm. After washing twice with 1 mL of MES buffer to remove excess EDC·HCl and NHSS, the magnetic nanoparticles were resuspended in 1 mL of PBS buffer (10 mmol/L, pH 7.4). Then, 40 μL of polyclonal antibodies against *Salmonella* (2.5 mg/mL) was added, and antibody conjugation was carried out at 37 °C for 2 h with shaking at 200 rpm to obtain the immunomagnetic nanoparticles (IMNPs). Unoccupied activated carboxyl sites on the IMNPs were blocked by incubation with 400 μL of skimmed milk solution (100 mg/mL in PBS buffer) at 37 °C for 3 h under shaking. After washing twice with 1 mL of PBS buffer, the IMNPs were stored in 1 mL of preservation buffer (PBS buffer supplemented with 5 mg skim milk powder, 10 mg BSA, and 250 mg sucrose) at 4 °C. The final concentration of antibodies incubated with the IMPNs was approximately 0.1 mg/mL.

### 2.3. Culture and Enumeration of Salmonella

*Salmonella* stored in 15% glycerol was taken out and allowed to equilibrate to room temperature, followed by gentle pipetting to achieve homogeneity. 100 μL of the revived bacterial suspension was inoculated into 5 mL of LB liquid medium, followed by incubation at 37 °C for 12 h in a thermostatic shaking incubator at 180 rpm to obtain the original bacterial culture, which was stored at 4 °C until use. Prior to the experiments, the original bacterial culture was serially diluted 10-fold with sterile PBS buffer to prepare bacterial suspensions with concentrations ranging from 1.0 × 10^1^ to 1.0 × 10^5^ CFU/mL.

For bacterial enumeration, the bacterial suspension to be counted was diluted to an appropriate concentration with sterile PBS buffer. 100 μL of the diluted suspension was evenly spread onto LB agar plates, with three replicate plates for each dilution. The plates were incubated at 37 °C for 16–18 h. After incubation, plates with less than 200 colonies were selected for counting. The initial bacterial concentration was calculated based on the number of colonies, dilution factor, and plating volume.

### 2.4. Development of the POCT System

**Figure 1:**
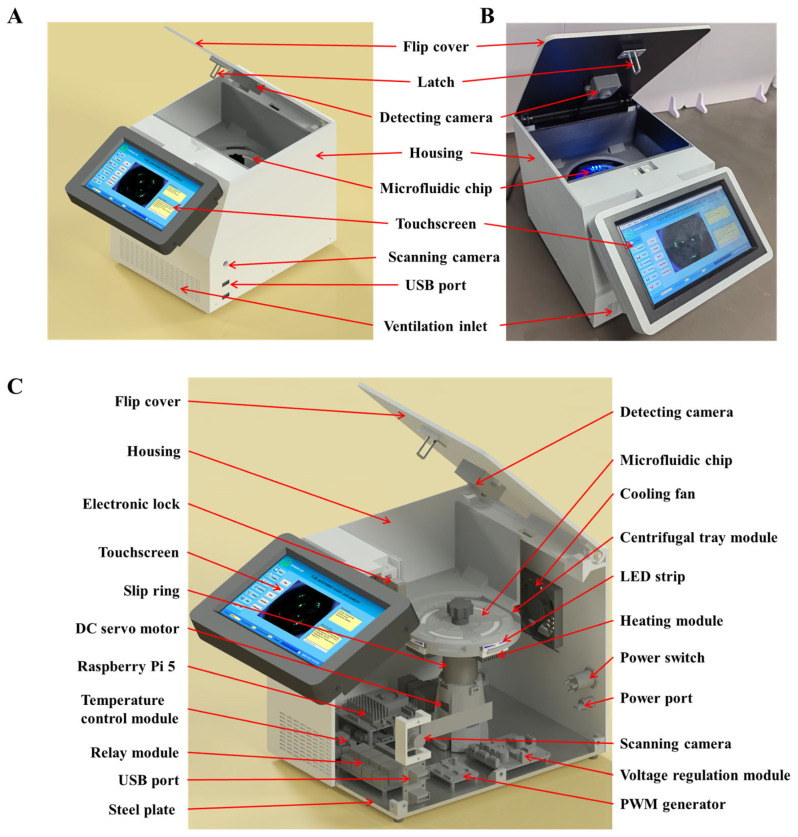
(**A**) Overall structural schematic diagram, (**B**) physical image, and (**C**) main hardware illustration of the POCT system based on the centrifugal microfluidic chip.

The schematic diagram for the overall structure of the POCT system is shown in Figure 1, with dimensions of 250 mm × 200 mm × 220 mm. The dynamic demonstration can be found in Video S1. A flip cover was located at the top of the system and fixed by an electronic lock, facilitating the placement and removal of the microfluidic chip to be tested. A 7-inch touchscreen was attached to the front of the system via a hinge and can be rotated freely, allowing users to adjust the orientation of the display screen. Ventilation inlets were arranged at the lower front of the housing, and a cooling fan was installed at the rear end, forming a reliable heat dissipation system. Two USB ports were provided on the right side of the housing for connecting external devices such as label printers and USB flash drives. A scanning camera for a quick response (QR) code was also fixed on this side to record sample collection information and chip information for convenient sample traceability. A power port and a power switch were located at the rear end of the housing for connecting a power adapter or battery with a voltage of 24 V to supply power for the system. A heavy steel plate was fixed at the bottom, and shock-absorbing soft pads were attached to the bottom surface to effectively reduce the vibration caused by the centrifugal rotation of the motor. The main hardware components were mounted on the steel plate (Figure 1C), including a Raspberry Pi 5 development board for central controller, a DC servo motor for providing centrifugal force for the microfluidic chip, a temperature control module for controlling the operation of Peltier chips and feeding back the temperature from the NTC-10K temperature sensor, a relay module for switch control of the electric control lock, light emitting diode (LED), temperature control module, etc., a pulse width modulation (PWM) generator for controlling the rotational speed of the motor, and a voltage regulation module for providing 5 V and 12 V voltages for each hardware module. Figure S1 shows the electrical connection flowchart of its main hardware.

The supporting human–computer interaction software is developed using NI LabVIEW 2023 Q1. Its main functions included (Figure S2): detection information entry and operation, data analysis, data saving and review; QR code printing of sampling information, single-step debugging of the control unit, and editing of the overall detection workflow.

### 2.5. Design of Centrifugal Microfluidic Chip

**Figure 2:**
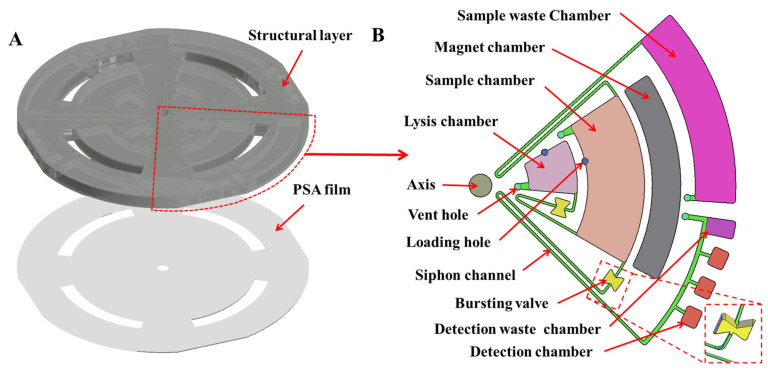
(**A**) Schematic diagram of the overall structural design of the centrifugal microfluidic chip and (**B**) the explanatory diagram of its detection unit (a partially enlarged 3D image showing the structure of the blasting valve).

The centrifugal microfluidic chip integrated operational steps of sample enrichment, sample lysis, nucleic acid amplification, and signal detection. The transfer of fluids on the chip was precisely controlled solely via a rotational speed gradient, without the need for manual intervention or external auxiliary equipment. As shown in Figure 2A, the microfluidic chip is disk-shaped, consisting of a transparent PSA film and a structural layer, with a maximum diameter of 100 mm and a thickness of 5 mm. Four sets of completely independent detection units capable of parallel detection were distributed on the structural layer, arranged in a centrally symmetric structure to maintain its balance during high-speed rotation. From the inside to the outside along the radial direction (i.e., the direction of centrifugal force), each unit consisted of one lysis chamber, one sample chamber, one magnet chamber, one sample waste chamber, one detection waste chamber, and three detection chambers (Figure 2B). The lysis chamber (~200 μL) was used to store nucleic acid release buffer and connected to the sample chamber via a siphon channel with a bursting valve in series. The sample chamber (~900 μL) was designed to hold the sample. It adopted a dual-channel structure: it was connected with the sample waste chamber through one siphon channel, and the detection chamber via a bursting valve and another siphon channel. The magnet chamber was a receiving slot for inserting a permanent magnet from outside the chip, providing a high-intensity magnetic field on the inner wall region of the sample chamber adjacent to the magnet for magnetic separation and enrichment of the target sample. The sample waste chamber (~1100 μL) was pre-embedded with absorbent cotton to collect waste fluid flowing from the sample chamber. The detection chamber (~35 μL) was pre-loaded with lyophilized microspheres for nucleic acid amplification and fluorescence signal detection (Figure S3). In addition to the relevant enzymes and dNTP mixture required for MIRA, these lyophilized microspheres also contained the primers and fluorescent probes, whereas the Mg^2+^ containing buffer for activating enzyme activity was added into the nucleic acid release buffer. Adjacent to the detection chamber was a detection waste chamber for storing excess sample nucleic acid reagents. Both the lysis and sample chambers were designed with an injection hole for injecting relevant reagents. All chambers were designed with vent holes to ensure that each chamber was exposed to the atmosphere to avoid negative pressure.

To achieve the integration of multiple operational steps, the centrifugal microfluidic chip required various microvalves for the complex control of multi-stage fluids. Among them, the microvalve formed by a siphon channel was often adopted. The siphon channel enabled directional fluid flow through the dynamic balance between capillary force and centrifugal force. At low rotational speeds, capillary force dominated in the hydrophilic channel, driving the fluid over the crest into the downstream chamber. At high rotational speeds, centrifugal force suppressed fluid flow and prevented premature transfer. In short, by regulating the rotational speed (i.e., static priming, high-speed inhibition, and low-speed release), the siphon channel allowed precise control of stepwise fluid transfer without external actuation, greatly simplifying the design of the microfluidic chip and reducing costs.

However, the siphon channel had two significant drawbacks. As shown in Figure 2B, to overcome the drawback that the siphon channel was prone to unexpected actuation when its inlet contacted the fluid, a bursting valve was connected in series to act as a physical barrier between them. The bursting valve was a passive microfluidic valve to hinder fluid flow by utilizing the abrupt change in capillary force caused by the geometric variation in the microchannel. It could be “broken through” only when the external driving pressure exceeded a critical value (burst pressure threshold). To achieve a high burst pressure threshold and thus improve the controllability and stability of the serially connected siphon channel, the bursting valve was optimally designed. The bursting valve adopted a dumbbell-shaped structure, with obtuse chamfers (120°) at both the inlet and the outlet. Its depth was slightly deeper than that of the siphon channel, and its inner wall was hydrophobically treated, which increased the difficulty of spontaneous filling of the bursting valve by fluid (as shown in the enlarged partial view in Figure 2B). Before the siphon channel was used, its inner wall was hydrophilically treated to provide a sufficiently strong capillary force for proper actuation. However, when the siphon channel transferred the fluid, its surface hydrophilicity decreased due to washing, or bubbles might enter the channel, making it difficult to reactivate stably for subsequent fluid delivery. To address this drawback, a dual-channel structure was designed (e.g., in the sample chamber of the microfluidic chip), so that two kinds of fluids were transferred by two independent siphon channels, respectively. To ensure that the siphon channel connecting the sample chamber and the sample waste chamber exhibits reduced hydrophilicity and fails to reactivate after transferring the supernatant of the bacterial sample captured by the magnet, the channel was treated with a hydrophilic reagent diluted 20 times with isopropanol. Thus, the subsequent target nucleic acid in the sample chamber was transferred to the detection chamber through another siphon channel. The innovative design of the bursting valve and dual-channel structure not only simplified the design of the microchannels and chambers of the chip but also reduced the fabrication cost of the chip while ensuring its stability.

### 2.6. Design of Centrifugal Tray Module

**Figure 3:**
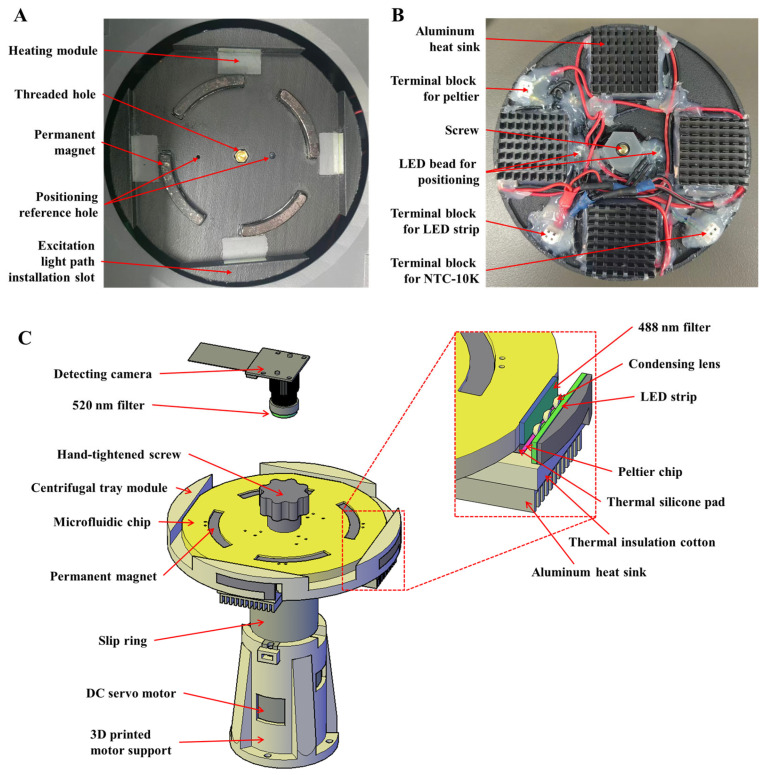
Physical structure of the centrifugal tray module: (**A**) front side, (**B**) back side, and (**C**) schematic of the detection optical path and thermostatic control module installed on it (partial enlarged image hides the centrifugal tray module).

The centrifugal tray module was equipped with the thermostatic control module and the fluorescence detection module for nucleic acid amplification and detection. As shown in Figure 3, the centrifugal tray module adopted a circular disk-shaped structure, consisting of a threaded hole, four permanent magnets, two positioning reference holes, four excitation light path installation slots, and four thermostatic control modules. The threaded hole was used to install the microfluidic chip and fixed with thumb screws. Permanent magnets were embedded in the surface of the centrifugal tray module to provide a magnetic field for immunomagnetic separation in the microfluidic chip and served as positioning pins to ensure the accurate installation of the microfluidic chip. The positioning reference holes consisted of one large hole and one small hole, and each hole was integrated with an LED bead to generate a light spot captured by the fluorescence acquisition module. These holes served as the origin and directional reference of the image coordinate system, enabling precise positioning of the detection chambers on the chip. The excitation light path installation slots were located on the positive side of the detection chambers of the microfluidic chip after installation of the excitation light path modules. The thermostatic control modules were fixed on the front surface of the excitation light path modules. The power supply for the LED beads, the excitation optical path modules, and the thermostatic control modules were all delivered through a conductive slip ring mounted beneath the centrifugal tray module. Each excitation optical unit consisted of an LED strip (including three LED beads), three condensing lenses, and an excitation filter arranged coaxially and sequentially along the optical path. The excitation light emitted by the LED strip was focused by a condenser lens to increase the excitation intensity density, and then filtered by a 488 nm filter to remove stray light. The excitation optical path remained stationary relative to the microfluidic chip, ensuring that the excitation light always acted precisely on the detection chambers. Each detection chamber was individually irradiated by one LED from the lateral side, which yielded low background reflection and allowed simultaneous excitation of multiple detection chambers by LEDs. A detecting camera was fixed directly above the centrifugal tray module, orthogonal to all excitation light paths, to capture the regions of all detection chambers simultaneously. A fluorescence acquisition filter mounted at the lens entrance of the detecting camera was used to effectively filter out stray excitation light and ambient light, thereby improving the signal-to-noise ratio (SNR) of the fluorescence signals.

The thermostatic control modules were installed on the centrifugal tray module directly beneath the detection chambers of the microfluidic chip, and each included a thermal silicone pad, an NTC-10K temperature sensor, a Peltier chip, thermal insulation cotton, and an aluminum heat sink. The thermal silicone pad possessed adhesiveness and elasticity, which could efficiently conduct the heat generated by the underlying Peltier chip. To ensure tight contact between the detection chambers and the thermal silicone pad, dimethyl silicone oil was applied onto the pad when installing the microfluidic chip. The NTC-10K temperature sensor was embedded in the thermal silicone pad to provide real-time temperature feedback to the temperature control module. The temperature was automatically controlled by the Peltier chip, with a temperature control accuracy of less than 0.1 °C. To reduce temperature fluctuations, the periphery of the Peltier chip was wrapped with thermal insulation cotton, and its bottom surface was closely attached to the heat-dissipating aluminum block.

### 2.7. MIRA Reaction and Fluorescence Detection

To be suitable for the microfluidic chip of the developed POCT system, the MIRA reagents were customized and modified by the manufacturer. The MIRA reagents were prepared in the form of lyophilized microspheres, which could be conveniently pre-embedded in the detection chambers of the microfluidic chip and stored for a long time at 4 °C after vacuum packaging. Each lyophilized microsphere contained various functional enzymes (recombinase, single-stranded DNA binding protein, DNA polymerase, etc.), dNTP mixture, primers, and fluorescent probes. In our platform, specific primers and fluorescent probes were adopted to target the conserved *invA* gene of *Salmonella*. As a classic specific biomarker, the *invA* gene exhibits excellent specificity and has been widely applied in the rapid detection of *Salmonella*. The buffers containing Mg^2+^ that could affect the activity of functional enzymes were preloaded in the nucleic acid release agents and transferred to the detection chambers along with the nucleic acid templates to dissolve the MIRA lyophilized microspheres. The temperature control module immediately regulated the Peltier chips to maintain a constant temperature of 42 °C for the MIRA reaction. At the same time, all LED strips were activated to excite the fluorescent probes in the detection chambers, and real-time fluorescence signals were collected synchronously by a detection camera every 30 s. The data processing software used two image positioning reference points in each fluorescent image as directional benchmarks to establish a coordinate system for the precise positioning of all detection chambers (Figure S4).

The grayscale values of each detection chamber across all fluorescence images were first extracted as fluorescence signals. Nucleic acid amplification curves, plotting fluorescence signals against amplification time, were generated. Then, the software applied Savitzky–Golay filtering and baseline subtraction to refine these curves. When the fluorescence signal reached a predefined fluorescence threshold, the corresponding amplification time was designated as its time threshold. Finally, a linear calibration curve relating bacterial concentrations to these time thresholds was constructed for quantitative analysis.

### 2.8. Sample Detection Process

**Figure 4:**
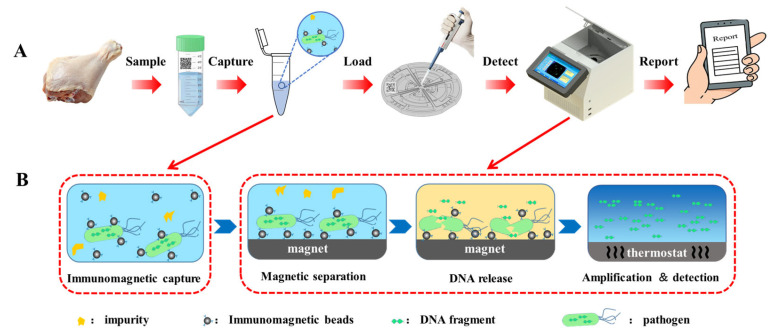
(**A**) The sample detection operation flowchart based on the POCT system and (**B**) the explanatory diagram of the bacterial detection method.

Figure 4 schematically illustrates the sample detection operation flowchart and bacterial detection method based on the POCT system. Prior to detection, an appropriate amount of immunomagnetic nanoparticles (IMNPs) was added to the bacterial sample and incubated in a shaking environment to achieve effective capture of bacteria. Before conducting detection using the POCT system, the QR codes on the sample and microfluidic chip were respectively scanned by the scanning camera, and their relevant information was accurately entered into the POCT system. Then, the system automatically matched the pre-set and optimized detection procedure to ensure that the subsequent detection was carried out in the designated steps. A total of 750 μL of the sample was loaded into the sample chamber, and 150 μL of nucleic acid release agent was injected into the lysis chamber of the microfluidic chip. The loading holes were sealed with adhesive tape to prevent sample leakage or external contamination. Subsequently, the microfluidic chip was mounted and fixed on the centrifugal tray module, and the flip cover of the system was closed. Finally, the system was started to automatically carry out the detection work according to the pre-set detection procedure. 

During the detection process of the system, the system first sequentially completed key steps such as magnetic separation of bacteria in the sample, bacterial lysis, and nucleic acid extraction through gradient centrifugation. After nucleic acid extraction was completed, the extracted nucleic acid templates were transferred to the detection chambers of the microfluidic chip. Subsequently, the temperature of the detection chambers was precisely controlled at 42 °C by the temperature control module to perform the MIRA reaction. At the same time, all LED light sources were activated, and the camera was used to continuously acquire fluorescence images of all detection chambers at a frequency of one photograph every 30 s to monitor the reaction process and results in real time. After the completion of all sample detection, the system automatically saved the relevant data, including fluorescence image test results, sample information, and microfluidic chip information, for subsequent further data analysis and traceability. The entire bacterial detection process was efficient and rapid, and completed in 60 min, including 25 min for immunomagnetic capture of bacteria and approximately 30 min for bacterial detection on the system.

## 3. Results

### 3.1. Working Principle and Process of Microfluidic Chip

**Figure 5:**
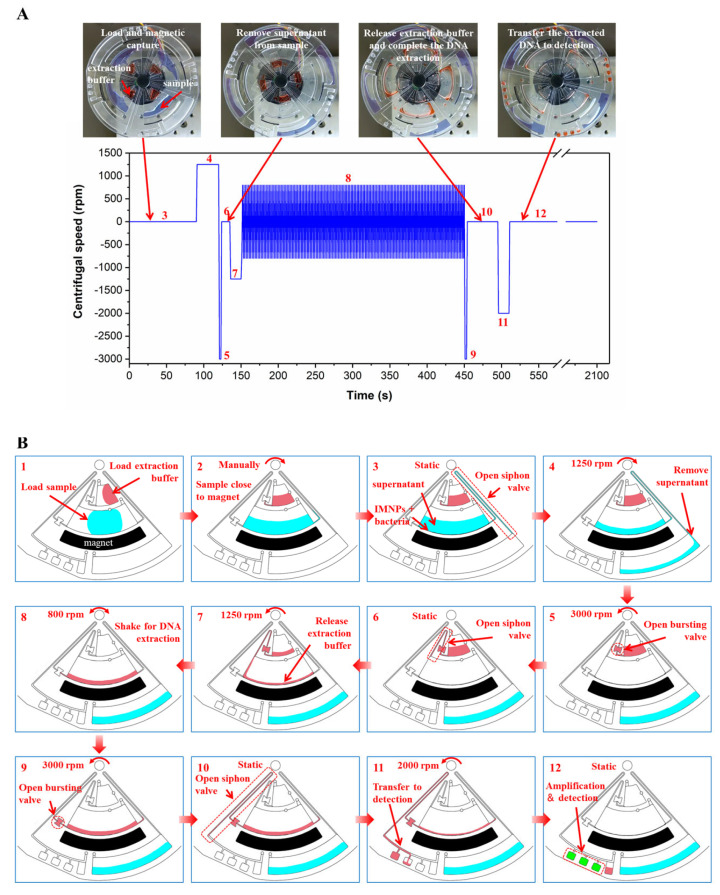
(**A**) Control process for the centrifugal motor and (**B**) workflow of the microfluidic chip at different stages.

This centrifugal microfluidic chip could precisely and sequentially trigger the pressure-activated opening of bursting valves and the effectiveness of siphon channels on the chip by controlling the rotational speed gradients of the centrifugal motor (i.e., stop, low speed, medium speed, and high speed). In this way, it drove different fluids to transfer among various chambers along predetermined paths, enabling the automatic completion of a series of operations of sample enrichment, nucleic acid extraction, nucleic acid detection, and so on, without the need for any external pumps, valves, or manual intervention. The specific gradient centrifugation control process of the motor is illustrated in Figure 5A. The symbols “+” and “−” in the figure indicated the direction of motor rotation, where “+” represented clockwise rotation and “−” represented counterclockwise rotation. Since the camera of the POCT system captured images in a dark chamber equipped with a filtering system, it could not observe the dynamic transfer process of non-fluorescent fluid in the microfluidic chip. Therefore, a simple centrifugal control system was established to simulate the centrifugation steps in actual POCT operation, and a smartphone was used to continuously record the entire fluid transfer process in the microfluidic chip (Figure S5). To enhance visualization, ink was used instead of colorless and transparent fluid. The dynamic demonstration can be found in Video S2.

The detection workflow of the microfluidic chip is illustrated in Figure 5B. Through the loading holes, the sample (750 μL) was injected into the sample chamber of the microfluidic chip, the nucleic acid lysate (150 μL) was injected into the lysis chamber, and the loading holes were sealed with tape. The microfluidic chip was mounted on the motor axis and manually rotated so that the sample in the sample chamber approached the magnet and contacted the inlet of the siphon channel. After standing for 90 s, the IMNP–bacteria complexes of the sample were captured on the inner wall near the magnet under the action of the magnetic field. Meanwhile, the siphon channel connected to the sample waste chamber was actuated by capillary force. The bursting valve connected to the detection chamber on the other side was not ruptured, so the siphon channel in series remained inactive. The motor was rotated clockwise at a low speed (1250 rpm) for 30 s, so that the supernatant in the sample chamber was transferred to the sample waste chamber, thus completing sample enrichment. After that, the siphon channel was interrupted by bubble entrapment, and the hydrophilicity of its inner wall decreased. The motor was immediately switched to high-speed counterclockwise rotation at 3000 rpm for 3 s, making the nucleic acid lysis buffer in the lysis chamber break through the bursting valve connected to the sample chamber, and conducting the series-connected siphon channel during the subsequent process of stopping centrifugation for 12 s. The motor was then rotated counterclockwise at 1250 rpm for 30 s to transfer the nucleic acid lysis buffer into the sample chamber. Subsequently, the motor was set to reciprocating rotation (clockwise and counterclockwise alternating once per second) at 800 rpm for 5 min, allowing sufficient contact between the nucleic acid lysis buffer and the IMNP–microbe complexes captured on the inner wall of the sample chamber. During this process, the bacteria broke down and released nucleic acids. At the same time, the siphon channel connected to the sample waste chamber could not be reactivated due to the decline of hydrophilicity and the presence of bubbles, while the siphon channel connected to the detection chamber also remained inactive because its in-series bursting valve could not be ruptured under low-speed centrifugation. After the nucleic acid was released, the motor was rotated counterclockwise at 3000 rpm for 3 s, so that the bursting valve connected to the detection chamber was broken, and the siphon channel in series was turned on during the subsequent process of stopping centrifugation for 42 s. The motor was then rotated counterclockwise at 2000 rpm for 15 s, allowing the nucleic acid in the sample chamber to sequentially fill the detection chambers, with the excess flowing into the detection waste chamber. Finally, the pre-loaded lyophilized reagents for nucleic acid amplification in the detection chambers were dissolved, and the nucleic acid amplification reaction and concentration detection were carried out at the specific temperature.

### 3.2. Optimization of Conditions for Immunomagnetic Separation

Immunomagnetic separation could improve the sensitivity of bacterial detection and remove interfering substances through specific capture to enhance the detection specificity and accuracy. Efficient capture of target bacteria from the sample background using IMNPs was a prerequisite for implementing this technology. A total of 750 μL of *Salmonella* (1.0 × 10^5^ CFU/mL) was taken, mixed with an appropriate amount of IMNPs, and incubated on a constant temperature shaker (37 °C, 200 rpm) for a period to complete bacterial capture. After magnetic separation, the captured bacterial precipitate and the uncaptured supernatant were obtained. The bacterial precipitate was resuspended with 750 μL of PBS buffer, diluted 10 times with 10 μL, and spread on the agar plate. Similarly, 10 μL of the uncaptured supernatant was taken, diluted 10-fold, and spread on another agar plate. After overnight incubation at 37 °C, the ratio of the number of colonies in the bacterial precipitate to the sum of the number of colonies in the bacterial precipitate and the supernatant plate was calculated as the efficiency of the immunomagnetic capture. In this study, the dosage of IMNPs and the immune reaction time were optimized, and the efficiency of immunomagnetic capture under different conditions was compared. Colony culture agar plate images are shown in Figures S6 and S7.

**Figure 6:**
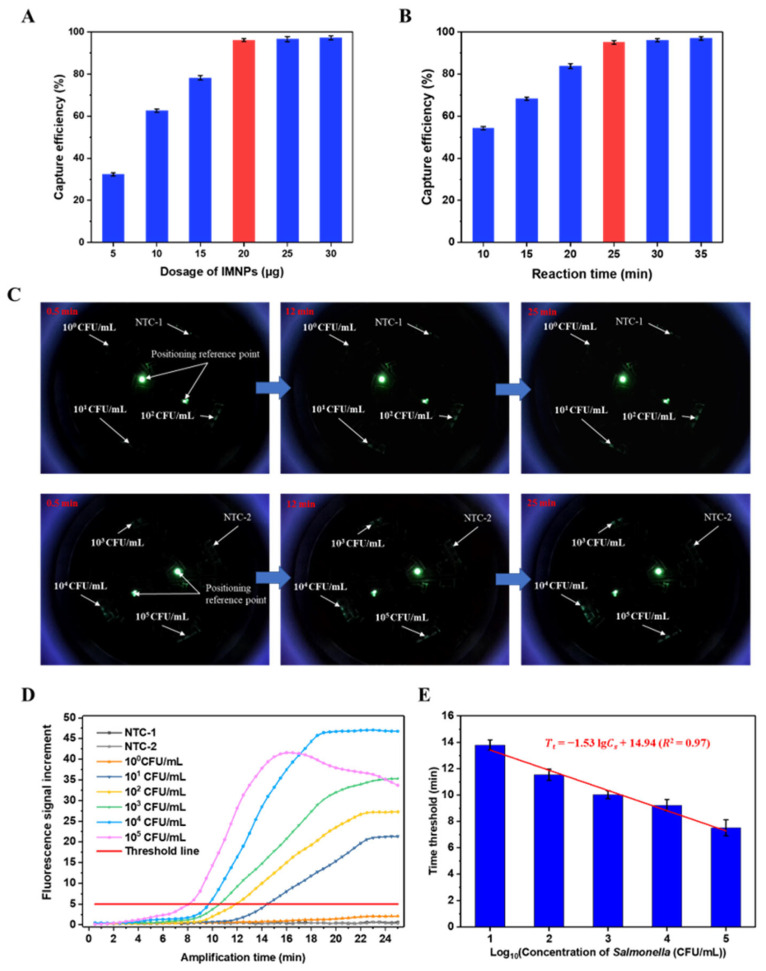
Optimization of IMNPs dosage (**A**) and reaction time (**B**) for capturing *Salmonella*. (**C**) Fluorescence detection images, (**D**) time-sequence plots of fluorescence detection signals, and (**E**) calibration curve plots for the detection of *Salmonella* at different concentrations using the POCT system (*N* = 3).

Different dosages of IMNPs, ranging from 5 μg to 30 μg, were used to perform immunomagnetic capture of *Salmonella* for 35 min, respectively. As shown in Figure 6A, with the increase in the dosage of IMNPs from 5 μg to 20 μg, the capture efficiency of target bacteria increased from 32.5% to 96.9%. As the dosage of IMNPs further increased, the capture efficiency of target bacteria did not increase significantly, indicating that the dosage of IMNPs was basically sufficient to capture all target bacteria at this time. Therefore, 20 μg was used as the optimal dosage of IMNPs for capture in this study.

A total of 20 μg of IMNPs were used to perform immunomagnetic capture of *Salmonella* with different reaction durations ranging from 10 to 35 min. As shown in Figure 6B, as the immune reaction time was extended from 10 min to 25 min, the capture efficiency of the target bacteria increased significantly from 53.6% to 95.5%. A further extension of the reaction time did not result in a notable improvement in capture efficiency, indicating that the IMNPs had already completed the capture reaction with the target bacteria sufficiently. Therefore, 25 min was determined as the optimal reaction time for immunomagnetic capture in this study.

### 3.3. Performance of POCT System

To evaluate the performance of the POCT system for detecting unknown concentrations of *Salmonella* in samples, different concentrations of *Salmonella* ranging from 1.0 × 10^1^ to 1.0 × 10^5^ CFU/mL were detected under optimal conditions, with sterile PBS buffer used as the no-template control (NTC) in each run to monitor for potential contamination (Figure 6C). Amplification curves over time were obtained to establish calibration curves between different bacterial concentrations ($C_{s}$) and their respective time thresholds ($T_{t}$). Videos S3 and S4 demonstrated the entire fluorescence detection process captured by the detection camera of the POCT system. As shown in Figure 6D, when the concentration of *Salmonella* decreased from 1.0 × 10^5^ to 1.0 × 10^1^ CFU/mL, the time threshold increased from 7.50 to 13.78 min. The amplification curve of *Salmonella* at a concentration of 1.0 × 10^5^ CFU/mL exhibited a decrease in signal intensity after reaching the plateau phase. This phenomenon was attributed to the accumulation of byproducts generated during the amplification reaction of high-concentration nucleic acid templates, which inhibited the activity of DNA polymerase and resulted in reduced amplification efficiency. The detection sensitivity was as low as 10 CFU/mL, indicating that the POCT system had a high detection sensitivity. As shown in Figure 6E, there is a good linear relationship between the time threshold and the logarithm of *Salmonella* concentration, with a determination coefficient (R^2^) of up to 0.97. The expression is shown in the following equation:

(1) $$T_{t}=-1.53\log C_{s}+14.94$$

To verify the specificity of the POCT system, four common non-target bacteria at 1.0 × 10^3^ CFU/mL, including *E. coli* O157:H7, *Staphylococcus aureus*, *Vibrio parahaemolyticus*, and *Listeria monocytogenes*, were tested. As shown in Figure S8, the experimental results confirmed that no false-positive amplification signals were observed for these non-target bacteria and no-template controls, demonstrating the good specificity of the POCT system. This benefits from the dual recognition mechanism of the system: the immunomagnetic nanoparticles are modified with polyclonal antibodies against *Salmonella*, which can effectively separate *Salmonella* from most non-target bacteria during immunomagnetic separation. Meanwhile, the MIRA kit specifically targets the conserved *invA* gene of *Salmonella*, further ensuring the specificity of the detection.

To further clarify the analytical positioning of the proposed platform, representative microfluidic systems that integrate on-chip nucleic acid amplification are compared in Table S2. Highly integrated systems often rely on multilayer chips, valves, actuation, temperature control, and optical modules, increasing complexity and cost. In contrast, our centrifugal platform used a siphon/bursting-valve-based workflow to integrate immunomagnetic separation, nucleic acid release, MIRA, and fluorescence detection, achieving high-sensitivity and rapid detection of *Salmonella* while maintaining a simple and portable device configuration.

### 3.4. Detection of Salmonella in Milk Samples

To verify the applicability of the developed POCT system for detecting *Salmonella* in real food samples, skimmed milk purchased from a local supermarket was selected as the research model. According to the National Food Safety Standard of China, 25 mL of skimmed milk was diluted 10-fold with 225 mL of sterile PBS buffer to reduce background matrix interference. Five portions of the above sample (9 mL each) were taken, and 1 mL of *Salmonella* with different concentrations was added to each portion to prepare spiked milk samples containing 1.0 × 10^1^–1.0 × 10^5^ CFU/mL. In addition, another 10 mL of *Salmonella*-free sample was taken as the negative control group. Finally, the POCT system was employed to detect *Salmonella*, and each sample was analyzed in triplicate. The average recoveries and RSDs were calculated. As shown in Table 1, the recoveries of spiked bacteria at different concentrations ranged from 83.22% to 127.60%, with RSDs not exceeding 13.7%, demonstrating that the POCT system was applicable for the actual detection of *Salmonella* in skimmed milk.

## 4. Discussion

In this study, aiming at the core challenges faced by the traditional detection methods in the rapid detection of foodborne pathogens, such as low sensitivity, complex operation, and difficulty in on-site deployment, a sample-to-answer POCT system based on centrifugal microfluidic chips was successfully developed. Through the innovative integration of immunomagnetic separation, isothermal nucleic acid amplification, and a microfluidic chip, this system formed a fully automated analytical platform integrating target enrichment, nucleic acid amplification, and signal detection. Experimental results demonstrated that the system enabled high-sensitivity and rapid detection of *Salmonella* in spiked milk samples, with a practical detectable level of 10 CFU/mL and a total assay time of less than 1 h. It also exhibited an excellent recovery (between 83.22% and 127.60%) and reproducibility (RSD ≤ 13.7%), providing a reliable tool for rapid screening of pathogens in complex matrices.

The greatest novelty of this work lies in the rational structural design of the centrifugal microfluidic chip and the overall system integration. The proposed microfluidic chip, which introduced bursting valves in series with siphon channels and a dual-channel shunting structure, effectively overcame the technical bottlenecks of unexpected actuation in multistage siphon channels and poor stability of single-channel fluid transfer for multiple reagents. These designs simplified the microchannel and chamber architecture and reduced the manufacturing cost. The relative positions between the independent excitation light paths of the optical detection system and the detection chambers did not change with the rotation of the microfluidic chip, which improved the detection repeatability and avoided the light leakage and fluorescence crosstalk between chambers. The orthogonal optical path with lateral excitation and filter combinations effectively reduced the interferences from substrate autofluorescence and stray light, thereby improving the signal-to-noise ratio. Moreover, it supported multi-channel parallel detection without expensive optical scanning or electric synchronous control, showing good portability and compatibility.

Future work will focus on reducing the cost and improving the integration, such as developing application-specific integrated circuit boards to replace discrete components. In addition, the detection throughput and multiplexing capability will be further expanded to achieve simultaneous detection of multiple pathogens in different food matrices. Combined with smartphone and Internet of Things technologies, cloud-based data management will be realized to support immediate interpretation, remote sharing of detection results, and big data monitoring for food safety, promoting the widespread application of this POCT system in resource-limited settings and on-site rapid detection scenarios.

## Figures and Tables

**Table 1:** Detection of *Salmonella* added to skimmed milk using the POCT system (*N* = 3).

The Spiked Concentration (CFU/mL)	The Detected Concentration ^#^ (CFU/mL)	Recovery (%)	RSD (%)
*Salmonella* free	Not detected	-	-
1.0 × 10^1^	12.76	127.60	13.7
1.0 × 10^2^	83.22	83.22	9.1
1.0 × 10^3^	943.24	94.32	9.6
1.0 × 10^4^	9406.48	94.06	6.8
1.0 × 10^5^	92,642.86	92.64	12.8

^#^ The mean value of three replicates.

## Data Availability

The dataset is not currently available for public sharing.

## Supplementary Materials

The following supporting information can be downloaded at: https://www.mdpi.com/article/10.3390/bios16060321/s1, Figure S1: Electrical connection flowchart of the main hardware of the POCT system; Figure S2: The main supporting human–computer interaction software include: (A) detection information entry and operation; (B) data analysis; (C) data saving and review; (D) QR code printing of sampling information; (E) single-step debugging of the control unit; and (F) editing of the overall detection process; Figure S3: Image of centrifugal microfluidic chip, pre-embedded with absorbent cotton and lyophilized microspheres for MIRA reaction; Figure S4: Typical fluorescence image captured by the detecting camera after MIRA reaction; Figure S5: The simple centrifuge control system used for shooting demonstration videos of microfluidic chip workflows; Figure S6: Colony culture agar plate images after capturing *Salmonella* with different amounts of IMNPs: the upper image shows the supernatant of uncaptured bacteria, while the lower image shows the precipitate of captured bacteria (*N* = 3); Figure S7: Colony culture agar plate images after capturing *Salmonella* with IMNPs under different reaction durations: the upper image shows the supernatant of uncaptured bacteria, while the lower image shows the precipitate of captured bacteria (*N* = 3); Figure S8: Fluorescence detection images and time-sequence plots of fluorescence detection signals to verify the specificity of the POCT system; Table S1: Comparison of the proposed platform with standard qPCR and representative isothermal amplification-based assays; Table S2: Comparison of representative microfluidic platforms integrating on-chip DNA amplification for pathogenic bacteria detection; Video S1: Video demonstration of POCT system; Video S2: Demonstration video of the microfluidic chip’s workflow controlled by the centrifuge motor; Video S3 and Video S4: Video of nucleic acid fluorescence detection on the microfluidic chip during isothermal amplification process.

## Abbreviations

The following abbreviations are used in this manuscript:

POCT	Point-of-care testing
WHO	World Health Organization
PCR	Polymerase chain reaction
ELISA	Enzyme-linked immunosorbent assay
LFIA	Lateral flow immunoassay
LAMP	Loop-mediated isothermal amplification
RAA	Recombinase-aided amplification
RPA	Recombinase polymerase amplification
MIRA	Multienzyme isothermal rapid amplification
LFD	Lateral flow dipstick
RSD	Relative standard deviation
QR	Quick response
LED	Light-emitting diode
PWM	Pulse width modulation
PSA	Pressure-sensitive adhesive
SNR	Signal-to-noise ratio
IMNPs	Immunomagnetic nanoparticles
CNC	Computer numerical control
NTC	No-template control
