# Supplementary material for: A Centrifugal Microfluidic Platform Integrating Immunomagnetic Separation and Isothermal Amplification for Rapid and High-Sensitivity Detection of Foodborne Pathogens
Source: Biosensors (Basel). 2026 Jun 2;16(6):321. doi: 10.3390/bios16060321 (PMC13297579; doi:10.3390/bios16060321)
Supplement: Supplementary file 1 [file biosensors-16-00321-s001.zip › Supplementary File.pdf]

A Centrifugal Microfluidic Platform Integrating Immunomagnetic Separation and Isothermal Amplification for Rapid and High-Sensitivity Detection of Foodborne Pathogens

Qingfeng Zheng [1,2], Zhun Zhuang [1,2], Hua Lei [1,2], Jianhan Lin [1,2,*], Hua Yang [1,3,*] and Ruibin Hu [1,*]

[1] Institute of Biotechnology, Xianghu Laboratory, Hangzhou 311231, China; zhengqingfeng@xhlab.ac.cn (Q.Z.); sy20243082075@cau.edu.cn (Z.Z.); leihua@xhlab.ac.cn (H.L.)
[2] College of Information and Electrical Engineering, China Agricultural University, Beijing 100083, China
[3] Zhejiang Academy of Agricultural Sciences, Hangzhou 310021, China
* Correspondence: jianhan@cau.edu.cn (J.L.); yanghua@zaas.ac.cn (H.Y.); huruibin@xhlab.ac.cn (R.H.)

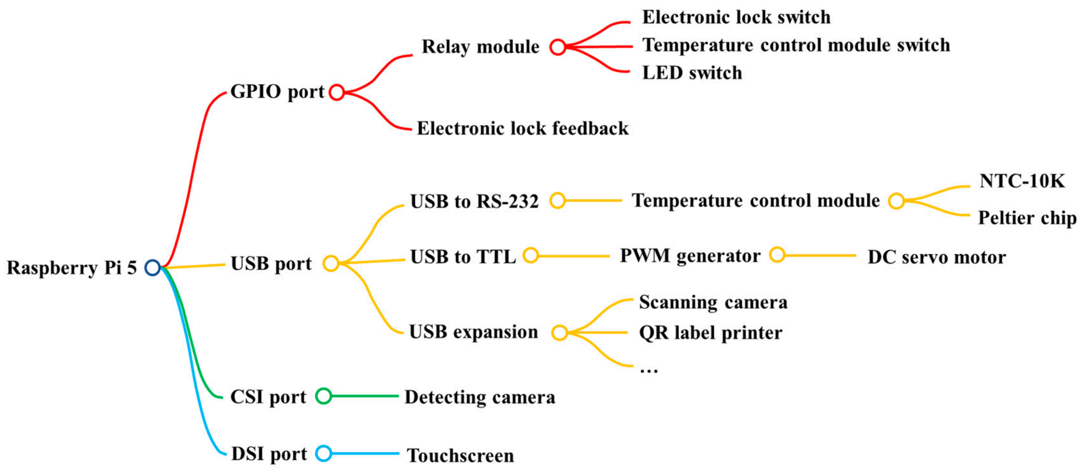

Figure S1. Electrical connection flowchart of the main hardware of the POTC system.

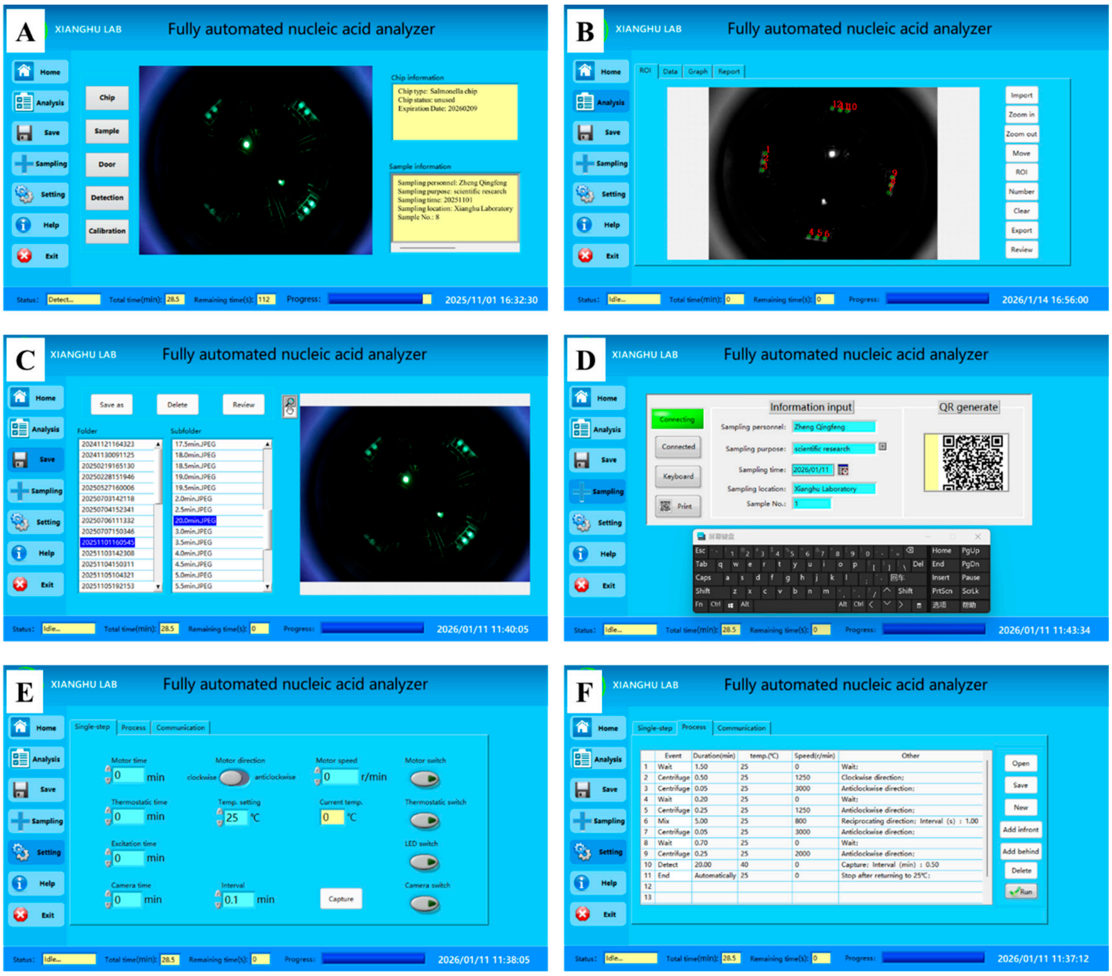

Figure S2. The main supporting human-computer interaction software include: (A) detection information entry and operation; (B) data analysis; (C) data saving and review; (D) QR code printing of sampling information; (E) single-step debugging of the control unit; and (F) editing of the overall detection process.

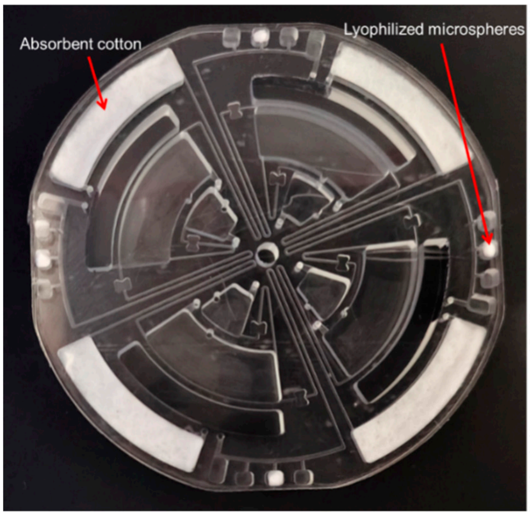

Figure S3. Image of centrifugal microfluidic chip, pre-embedded with absorbent cotton and lyophilized microspheres for MIRA reaction.

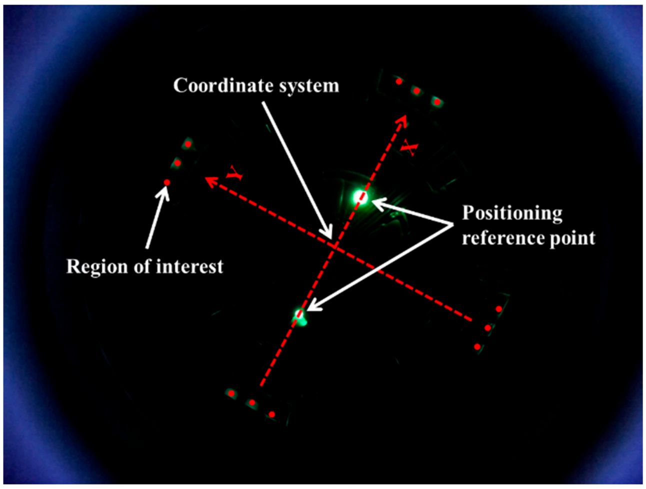

Figure S4. Typical fluorescence image captured by the detecting camera after MIRA reaction.

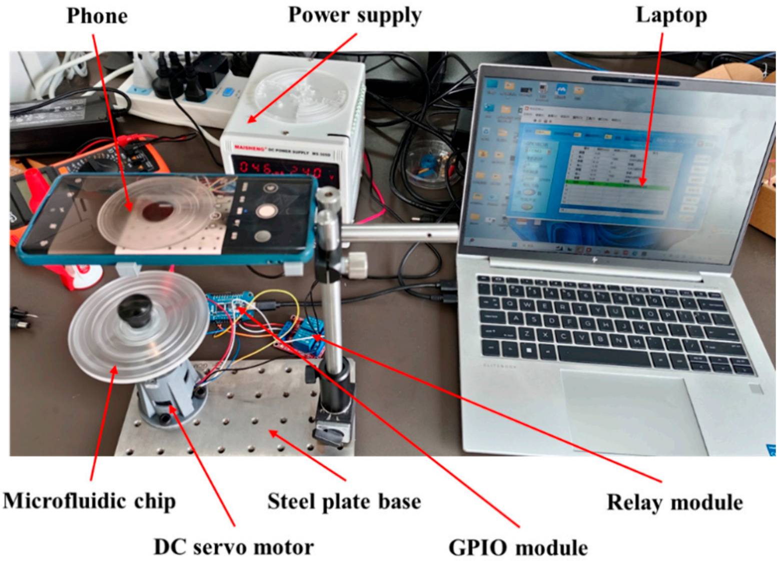

Figure S5. The simple centrifuge control system used for shooting demonstration videos of microfluidic chip workflows.

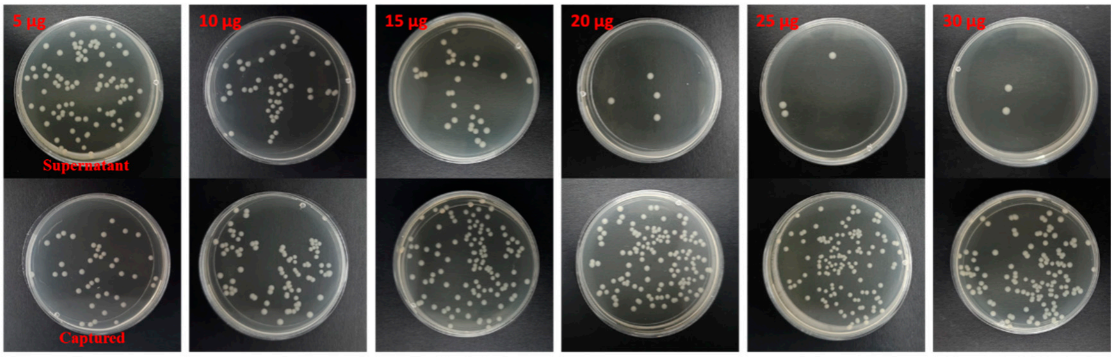

Figure S6. Colony culture agar plate images after capturing Salmonella with different amounts of IMNPs: the upper image shows the supernatant of uncaptured bacteria, while the lower image shows the precipitate of captured bacteria (N = 3).

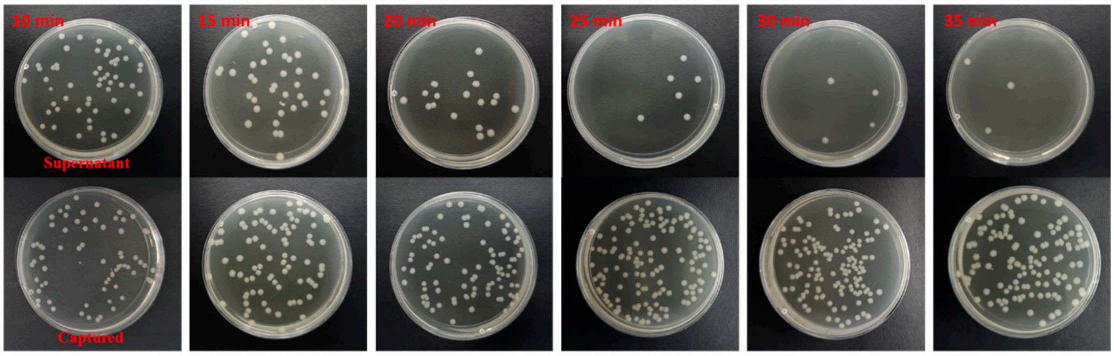

Figure S7. Colony culture agar plate images after capturing Salmonella with IMNPs under different reaction durations: the upper image shows the supernatant of uncaptured bacteria, while the lower image shows the precipitate of captured bacteria (N = 3).

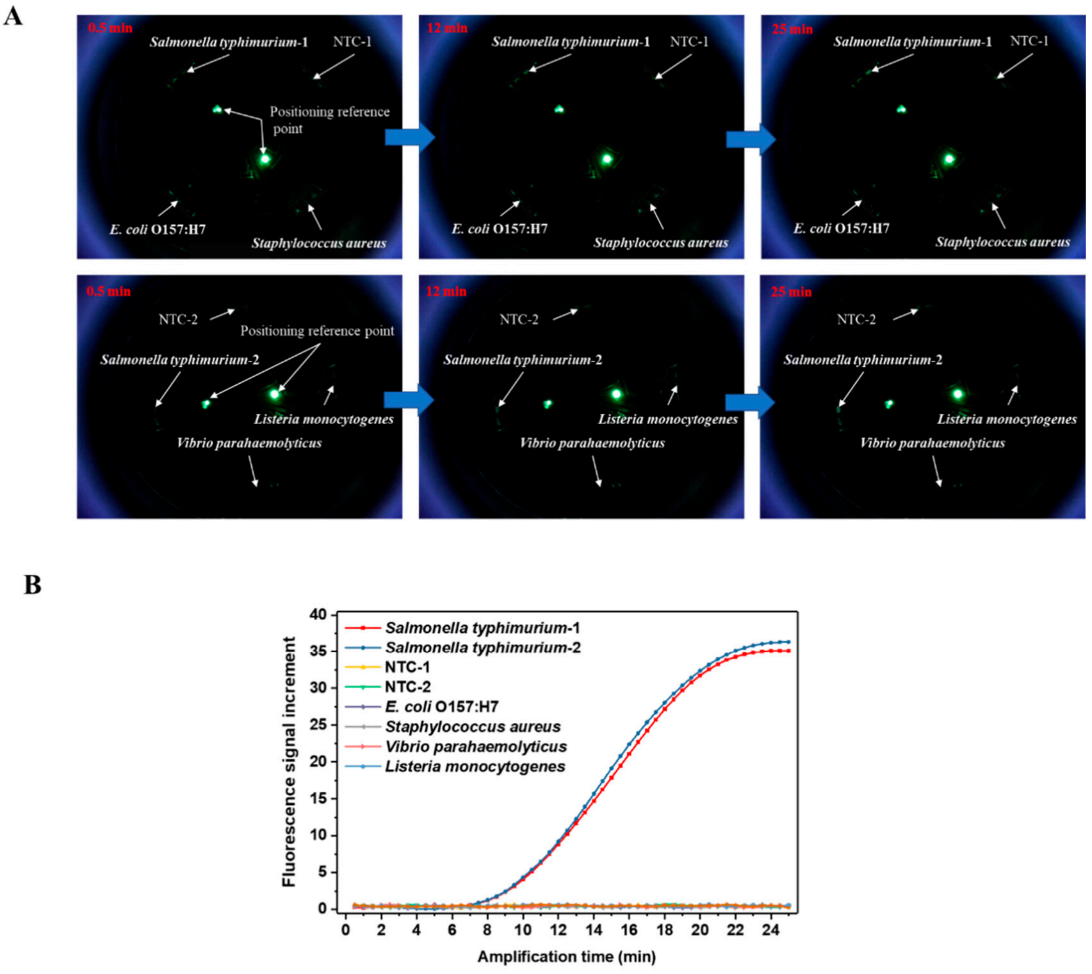

Figure S8. (A) Fluorescence detection images and (B) time-sequence plots of fluorescence detection signals to verify the specificity of the POCT system.

Table S1. Comparison of the proposed platform with standard qPCR and representative isothermal amplification-based assays

Method	Detection time	LOD / sensitivity	Sample preparation	Equipment needs	Assay cost	Ref
IMS-MIRA-FLD	<1 h	10 CFU/mL	IMNPs enrichment, lysis, MIRA amplification, on-chip fluorescence detection	Portable centrifugal POCT system	Low-moderate	This work
FDA BAM culture method	4-5 days	Presence/absence confirmation	Pre-enrichment, selective enrichment, selective plating, biochemical/serological confirmation	Incubator, biosafety cabinet, selective media, biochemical/serological tests	Low reagent cost; high labor/time cost	[1]
qPCR screening after pre-enrichment	Day 2 after 24 h pre-enrichment	positive results still require culture confirmation	Pre-enrichment and DNA extraction/template preparation	qPCR instrument and molecular laboratory workflow	High equipment and moderate-high reagent cost	[1]
LAMP-TtAgo centrifugal chip	17 min	1 CFU/mL	Fast nucleic acid extraction and one-pot LAMP-TtAgo reaction	Centrifugal microfluidic chip and fluorescence reader	Moderate-high	[2]
High-throughput centrifugal LAMP chip	60 min	10^{-2} CFU/mL	On-chip lysis, magnetic-bead extraction, LAMP	Portable centrifugal device with heating, magnetic mixing, fluorescence detection	Moderate-high	[3]

Method	Detection time	LOD / sensitivity	Sample preparation	Equipment needs	Assay cost	Ref
RPA microfluidic system	30 min	10^{-2} copies/uL	Purified DNA or RPA reagent loading	Microfluidic chip, heater, smartphone fluorescence module	Moderate	[4]
MIRA-LFD	<20 min	115 CFU/mL	Cellulose filter paper DNA extraction, MIRA, lateral-flow dipstick	Simple heater at 39 °C and visual strip readout	Low-moderate	[5]
IMS-LAMP-NALFS	90 min	10^{-2} CFU/mL	IMS enrichment, PMAxx treatment, LAMP, lateral-flow strip	Magnetic separator, constant-temperature heater, strip readout	Moderate	[6]

Table S2. Comparison of representative microfluidic platforms integrating on-chip DNA amplification for pathogenic bacteria detection

Target	Integrated components	Fabrication complexity	Detection principle	Assay time	Sensitivity	Ref
S. Typhimurium	IMNPs enrichment, lysis, MIRA amplification, fluorescence detection	Low-moderate; PSA/structural disc and portable centrifugal analyzer	IMNPs -MIRA fluorescence	<1 h	10 CFU/mL in spiked milk	This work
S. aureus	Fast nucleic acid extraction, LAMP, TtAgo cleavage, fluorescence readout	Moderate-high; centrifugal multi-channel chip with preloaded LAMP/TtAgo reagents	LAMP-TtAgo fluorescence	17 min	1 CFU/mL	[2]
E. coli	Lysis, magnetic-bead extraction, LAMP, fluorescence detection	High; multilayer centrifugal disc with valves, magnetic mixing, heating, and fluorescence detection	LAMP fluorescence	60 min	10^{-2} CFU/mL	[3]
S. Typhimurium	Mixing chamber, RPA chamber, versatile valve, smartphone fluorescence	Moderate; PDMS/glass chip, 3D-printed valve, and magnetic mixer	RPA fluorescence	30 min	10^{-2} copies/uL	[4]
S. Typhimurium	DNA extraction, RPA, micro-capillary electrophoresis	High; PMMA/PSA chip, heater, and micro-capillary electrophoresis module	RPA + electrophoretic readout	75 min	10^{-3} CFU/mL	[7]
Salmonella and E. coli	Bacterial lysis, nucleic acid capture, elution, distribution, LAMP	High; active/passive valves, pneumatic module, and silicon-membrane extraction	LAMP fluorescence	40 min	25 *Salmonella* cells; 40 *E. coli* cells	[8]
S. Typhimurium, E. coli O157:H7*, L. monocytogenes*	Magnetic DNA extraction, RAA, pneumatic check valves, rotating valve, LED fluorescence	Moderate; PDMS chip, 3D-printed valve, and finger-driven operation	RAA fluorescence	45 min	15, 48, and 38 CFU/mL, respectively	[9]
A. hydrophila	DNA extraction, MIRA reaction chamber, vibration mixing, ceramic heating, air-pump transfer	Moderate-high; active fluid handling and heating modules	MIRA fluorescence	40 min	10 CFU/mL	[10]
S. Enteritidis, E. coli O157:H7*, S. aureus*	Silica-membrane DNA extraction, LAMP, magnet-regulating valves	High; 3D-printed disc, magnetic valves, and silicone sealing pads	LAMP fluorescence	40 min	10 CFU/mL in spiked chicken supernatant	[11]

References

1. Bacteriological Analytical Manual (BAM) Chapter 5: Salmonella. Available online: https://www.fda.gov/food/laboratory-methods-food/bam-chapter-5-salmonella (accessed on 30 May 2026).
2. Wang, N.; Zhang, Q.; Liu, Y.J.; Huang, B.Y.; Man, S.L.; Ye, S.Y.; Ma, L. Sample-in-answer-out centrifugal microfluidic chip reaction biosensor powered by Thermus thermophilus Argonaute (TtAgo) for rapid, highly sensitive and multiplexed molecular diagnostics of foodborne bacterial pathogens. Chem Eng J 2024, 495, 153434.

https://doi.org/10.1016/j.cej.2024.153434.
3. Lu, S.Y.; Yang, Y.Z.; Cui, S.Q.; Li, A.Y.; Qian, C.; Li, X.Q. Integrated High-Throughput Centrifugal Microfluidic Chip Device for Pathogen Detection On-Site. Biosensors 2024, 14, 313. https://doi.org/10.3390/bios14060313.
4. Jin, Y.; Wang, J.Y.; Wang, Z.Q.; Xiong, P.; Cheng, J.N.; Xu, T.Y. An Integrated Microfluidic Biosensing System Based on a Versatile Valve and Recombinase Polymerase Amplification for Rapid and Sensitive Detection of Salmonella typhimurium. Biosensors 2023, 13, 790. https://doi.org/10.3390/bios13080790.
5. Yi, S.Q.; Zhou, N.L.; Ma, Y.; Yi, L.Z.; Shang, Y. Super-Fast Detection of Bacillus cereus by Combining Cellulose Filter Paper-Based DNA Extraction, Multienzyme Isothermal Rapid Amplification, and Lateral Flow Dipstick (MIRA-LFD). Foods 2025, 14, 454. https://doi.org/10.3390/foods14030454.
6. Li, Q.X.; Zhang, J.F.; Chen, X.X.; Jiang, T.T.; Lin, L.; Zhao, L.C. Real-time and visual detection of viable Salmonella in milk from remote pasture via IMS-LAMP-NALFS. Microchem. J. 2024, 197, 109732. https://doi.org/10.1016/j.microc.2023.109732.
7. Nguyen, V.D.; Sureshkumar, G.; Seo, T.S. Integrated microfluidic device of DNA extraction, recombinase polymerase amplification and micro-capillary electrophoresis for sample-to-answer detection of Salmonella Typhimurium. Sens. Actuators B Chem. 2025, 435, 137625. https://doi.org/10.1016/j.snb.2025.137625.
8. Xiao, Y.J.; Zhou, M.F.; Liu, C.G.; Gao, S.Y.; Wan, C.; Li, S.J.; Dai, C.X.; Du, W.; Feng, X.J.; Li, Y.W.; et al. Fully integrated and automated centrifugal microfluidic chip for point-of-care multiplexed molecular diagnostics. Biosens. Bioelectron. 2024, 255, 116240. https://doi.org/10.1016/j.bios.2024.116240.
9. Jin, N.N.; Yang, F.Z.; Zhang, X.Y.; Li, Y.B.; Lin, J.H. Sensitive detection of multiplex bacteria based on finger driven microfluidics and recombinase aided amplification. Biosens. Bioelectron. 2025, 287, 117750. https://doi.org/10.1016/j.bios.2025.117750.
10. Bai, Z.Z.; Zhang, Y.Y.; Li, C.; Han, J.; Wu, T.L.; Wang, C.; Zhang, Y.; Li, D.L. A portable microfluidic platform for on-site detection of Aeromonas hydrophila in aquaculture water using multi-enzyme isothermal rapid amplification. J. Hazard. Mater. 2026, 501, 140677. https://doi.org/10.1016/j.jhazmat.2025.140677.
11. Wang, S.Y.; Shen, S.Q.; Kang, S.M.; Yan, Z.; Wu, S.J.; Liu, N.; Wu, A.B. Magnet regulating valve reconstructed lab-on-a-disc POCT system. Biosens. Bioelectron. 2026, 294, 118185. https://doi.org/10.1016/j.bios.2025.118185.

.
